# X-ray absorption spectroscopy of scandium oxide polymorphs

**DOI:** 10.1039/d5ra06446e

**Published:** 2025-12-03

**Authors:** Daniel Duarte-Ruiz, Holger-Dietrich Saßnick, Fabiana Machado Ferreira De Araujo, Marie Gentzmann, Thomas Huthwelker, Caterina Cocchi

**Affiliations:** a Institute of Physics, Carl-von-Ossietzy Universität Oldenburg 26129 Oldenburg Germany caterina.cocchi@uni-jena.de; b Bundesanstalt für Materialforschung und -prüfung (BAM) Unter den Eichen 87 12205 Berlin Germany; c Paul Scherrer Institut, Swiss Light Source (SLS) 5232, Villigen Switzerland; d Friedrich-Schiller Universität Jena, Institute for Condensed Matter Theory and Optics 07743 Jena Germany

## Abstract

Scandium oxide (Sc_2_O_3_) is a rare-earth oxide with significant potential in key technological areas, but due to its limited supply a deep understanding of its characteristics in different crystalline phases is still missing. Here, we present a combined experimental and *ab initio* X-ray absorption spectroscopy investigation of Sc_2_O_3_ focusing on excitations from the O K-edge and the Sc L_2,3_-edge. While measurements are performed on a cubic sample, the most stable phase under ambient conditions, six different polymorphs are computed, including two high-pressure phases with a trigonal and monoclinic lattice in addition to the cubic phase, as well as three computationally predicted structures. Our analysis of the structural and electronic properties reveals significant similarities between the cubic polymorph and the high-pressure trigonal phase, while the monoclinic crystal exhibits distinct features. The spectra simulated for these similar phases from the solution of the Bethe–Salpeter equation show very good agreement with measurements. Additional comparison with results computed in the independent-particle approximation highlights the dominant role of electron–hole correlations in shaping the absorption features, particularly at the O K-edge, where a common pattern with the features of other sesquioxides is identified. Our findings offer new insight into the spectral fingerprints of Sc_2_O_3_ polymorphs, aiding *in situ* characterization and informing sustainable materials management.

## Abstract 2

First-principles analysis of Sc_2_O_3_ polymorphs reveals structural and electronic analogies that are useful to compute and analyze X-ray absorption spectra in comparison to experiments.

## Graphical abstract

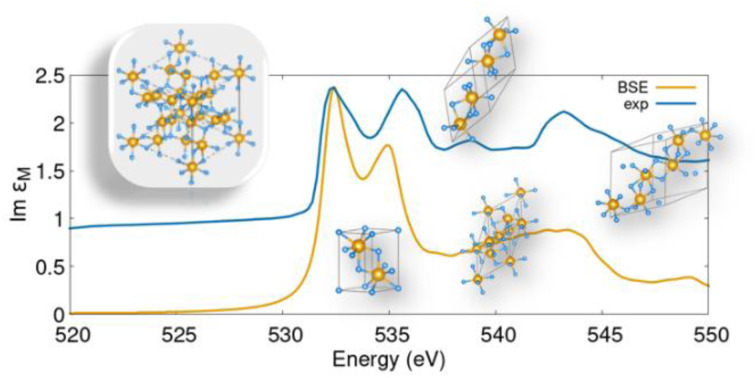

## Introduction

Scandium oxide (Sc_2_O_3_) is a technologically relevant material. Its high thermal stability and favorable optoelectronic properties, including a wide band gap,^1–4^ offer great potential for applications in solid oxide fuel cells, high-performance ceramics, photocatalysis, and gate dielectrics.^5–8^ The supply of this rare-earth oxide^9^ is limited in many developed countries, including the members of the European Union. The scarce availability of this material not only hinders its fundamental characterization but also underscores the growing importance of sustainable material management by detecting and identifying Sc_2_O_3_ in complex matrices from recycled materials or industrial waste.^10^

X-ray absorption near-edge structure (XANES) spectroscopy represents a powerful tool to probe the local structure and electronic characteristics of materials.^11^ XANES is particularly suited for identifying specific compounds in heterogeneous systems thanks to its element- and site-specific sensitivity, and its ability to resolve fine spectral features associated with different oxidation states and coordination environments.^12–19^ However, the accurate interpretation of XANES measurements is often challenged by the intrinsic polymorphism of many oxides, including Sc_2_O_3_. This compound can crystallize in multiple structures depending on the thermodynamic conditions during synthesis,^20–22^ and transitions among different crystal phases have been reported.^20–24^

A reliable theoretical approach complementing measurements is therefore essential for thorough material characterization. While the experimental community especially benefits from efficient and easy-to-use simulation tools like FDMNES^25^ and FEFF,^26^ methods based on (all-electron) density functional theory (DFT) and many-body perturbation theory (MBPT), including the solution of the Bethe–Salpeter equation (BSE),^27,28^ are considered the state-of-the-art approaches for the simulation of X-ray absorption spectra from first principles. Being formally independent of empirical parameters, they can be applied, in principle, to any material, including computationally predicted or metastable phases. Moreover, by explicitly accounting for electron–hole correlations, they provide fundamental insight into the key spectral characteristics that often dominate the signals.^29–35^ As such, these simulations have invaluably complemented XANES experiments and provided spectroscopic predictions of complex materials in the last two decades.^13,16,18,19,36–39^

The central objective of this work is to present a combined experimental and computational study that establishes a robust, high-accuracy diagnostic framework for the XANES signatures of Sc_2_O_3_ polymorphs. We perform absorption synchrotron measurements from the oxygen K-edge and Sc L_2,3_-edge on the most stable cubic phase, and complement these experiments with state-of-the-art *ab initio* calculations from DFT and MBPT on six Sc_2_O_3_ polymorphs. A detailed analysis of their structural and electronic characteristics lays the foundation for the subsequent analysis of the simulated XANES spectra. Given the significant computational expense of MBPT calculations, particularly for the large unit cells of the cubic (1) and orthorhombic (4) phases, we strategically select only four polymorphs for the demanding BSE calculations. This approach, which relies on the structural and electronic similarities identified in our dedicated analysis, allows us to maintain the required high accuracy for the core-level simulations while effectively providing high-fidelity spectral information for all six polymorphs. This strategy is validated by the excellent agreement between the experimental XANES and the BSE spectra calculated on smaller polymorphs. By contrasting these results against those obtained in the independent-particle approximation, where electron–hole interactions are neglected, we gain insight into the excitonic nature of the core-level excitations and reveal their importance, especially at the O K-edge. This comprehensive study provides a critical framework for the interpretation of experimental XANES spectra of Sc_2_O_3_ polymorphs, offering insights into their diverse local electronic and structural characteristics, crucial for advanced applications and sustainable resource management.

## Methodology

### Theoretical background and computational details

In the first step of our analysis, we optimize the geometries and analyze the structural properties of the considered Sc_2_O_3_ polymorphs using DFT as implemented in Quantum ESPRESSO.^40^ For these calculations, we adopt the Perdew–Burke–Ernzerhof (PBE)^41^ functional with cutoff values of 150 and 1200 Ry for plane-waves and density, respectively. In this framework, core electrons are represented *via* pseudopotentials taken from the pslibrary.^42^ 8 × 8 × 8 **k**-meshes are adopted to sample the Brillouin zone of all systems.

The electronic structure of the considered Sc_2_O_3_ phases is characterized using the all-electron full-potential code exciting,^43^ which implements density functional theory and the BSE for core-level spectroscopy.^44^ The BSE Hamiltonian

1 *Ĥ*^BSE^ = *Ĥ*^diag^ + 2*Ĥ*^x^ + *Ĥ*^dir^,

includes the diagonal term *Ĥ*^diag^, accounting for vertical transitions between core and conduction states, the repulsive exchange interaction *Ĥ*^x^, multiplied by 2 due to spin-degeneracy, and the direct term *Ĥ*^dir^, embedding the statically screened Coulomb interaction between electron and hole. This formalism uses unexcited, ground-state Kohn–Sham orbitals for both core and conduction states, with the electron–hole interaction terms (*Ĥ*^x^ and *Ĥ*^dir^) dynamically capturing the effects of the core–hole.^27^ As such, our BSE approach does not employ separate ground-state and final-state Hamiltonians like the ΔSCF or supercell core–hole methods. The effects of orbital hybridization, which are crucial in oxides like Sc_2_O_3_, are inherently included in the underlying Kohn–Sham states used as a basis to construct the BSE.

The diagonalization of the *Ĥ*^BSE^*via* the solution of the effective two-particle Schrödinger equation,2
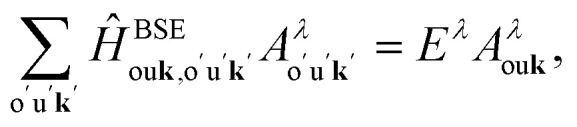


delivers the excitation energies *E*^*λ*^ as eigenvalues, while the eigenvectors *A*^*λ*^_ou**k**_ contain information about the oscillator strength and the composition of the *λ*^th^ excitation. XANES spectra are calculated from the imaginary part of the macroscopic dielectric function3
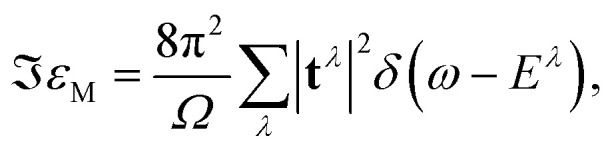
where *Ω* is the unit cell volume, *ω* is the angular frequency of the incoming radiation, and **t**^*λ*^ are the transition coefficients between the initially occupied (o) core levels and the unoccupied conduction states (u):4
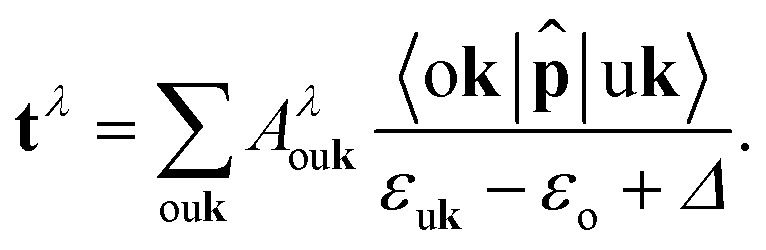


It is evident from eqn (4) that the BSE eigenvectors *A*^*λ*^_ou**k**_ weight the momentum matrix elements of the specific transition. The denominator, representing the Kohn–Sham energy difference between unoccupied and core states, is corrected by a scissors shift *Δ*, empirically determined by aligning the first maximum in the computed spectra with the one in the experimental reference, to amend the inherently underestimated core-level energies obtained from DFT. The same shift *Δ* is also applied in the independent-particle approximation (IPA), when electron–hole correlations are neglected, setting *H*^IPA^ ≡ *H*^diag^. In this simplified case, the solution of eqn (2) only yields a single *A*^*λ*^_ou**k**_ = 1 for each excitation *λ* (all the other eigenvectors are zero), corresponding, in the absence of Coulomb mixing, to a single vertical transition from the initial core level to a single unoccupied state at a given **k**.

In the linearized augmented plane-wave plus local orbital (LAPW + lo) method implemented in exciting,^43^ the basis set convergence is given by the product between the smallest muffin-tin radius among the considered atomic species and the plane-wave cutoff |**G** + **k**_MAX_|. By choosing the standard values *R*_MT_ = 1.8 bohr for Sc and *R*_MT_ = 1.6 bohr for O, we set 
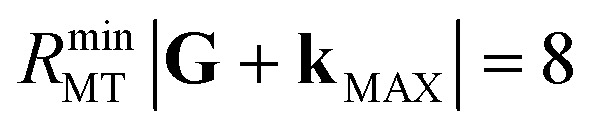
 for all considered crystals. The **k**-meshes employed to sample the Brillouin zones are listed in Table 1. XANES spectra from the O K-edge (Sc L_2,3_-edge) are simulated considering an energy window of 30 eV (40 eV) above the Fermi energy. A plane-wave cutoff of |**G**_MAX_| = 2 bohr^−1^ is employed for the Kohn–Sham response function of all considered polymorphs, while different **k**-meshes are adopted according to the unit-cell size; see Table 1. Scissors operators *Δ* = 104 eV and *Δ* = 24 eV, determined from our experimental data, are adopted for the XANES from the Sc L_2,3_-edge and the O K-edge, respectively. A Lorentzian broadening of 0.5 eV is applied to visualize all simulated spectra. This significantly larger value compared to the natural linewidths of Sc (∼0.2 eV) reported in the literature^45^ is empirically chosen to account for the shorter excitation lifetime and the occurrence of scattering events in Sc_2_O_3_ crystals.

**Table 1:** Space group, entry ID in the materials project (MP) database, crystal structure, and **k**-meshes adopted in the DFT and BSE calculations with exciting for the considered Sc_2_O_3_ polymorphs

No.	Space group	MP entry ID	Crystal structure	**k**-mesh (DFT)	**k**-mesh (BSE)
(1)	*Ia*3̄*c*	mp-216	Cubic	4 × 4 × 4	—
(2)	*R*3̄*c*	mp-755313	Trigonal	6 × 6 × 6	6 × 6 × 6
(3)	*C*2/*m*	mp-558748	Monoclinic	8 × 8 × 8	4 × 4 × 4
(4)	*Pna*2_1_	mp-775837	Orthorhombic	6 × 4 × 4	—
(5)	*R*3̄	mp-754455	Trigonal	7 × 7 × 7	5 × 5 × 5
(6)	*P*3̄*m*1	mp-13060	Trigonal	5 × 5 × 5	4 × 4 × 4

### Experimental methods

X-ray absorption spectra (XAS) were taken at the PHOENIX beamline^46^ at the Swiss Light Source (SLS). The PHOENIX II endstation was employed, which uses the optics of the X-Treme beamline.^47^ The source of the beamline is an elliptical undulator. Apple type II (period length 54 mm with 32 periods) monochromatic light is generated by a planar grating monochromator, adopting horizontal polarization throughout all experiments. The unfocused beam was shaped by a pinhole to a round beam of about 2 mm in diameter.

The Sc_2_O_3_ sample, produced in China and provided by the Dutch company KBM Affilips, was tested for purity with ablation-inductively coupled plasma mass spectrometry^48^ prior to the XAS measurements. The sample is a powder, which was mounted on a Cu plate by pressing it into a conducting carbon tape. The Cu plate was mounted electrically insulated and connected to an amperometer. The XAS measurement was performed in the endstation, which was held in a vacuum chamber at a pressure of about 10^−5^ mbar. The absorption spectra were measured in both total electron yield (TEY) mode and *via* partial X-ray fluorescence (XRF). The total current from the sample measured by a current amplifier (manufacturer: Keithley, USA) serves as the TEY signal. An energy dispersive silicon drift detector (manufacturer: Ketek, Germany) measures the partial fluorescence signal.

The endstation vacuum is separated from the beamline vacuum by a silicon nitride window of 0.5 µm thickness, which also contains nitrogen. This induces an N K-edge signal in the spectrum of the incoming photons, which overlaps with the scandium L-edge. Therefore, the intensity of incoming photons, *I*_0_, was measured on a clean Cu plate, located on the sample holder, downstream of the entry window. Taking advantage of the stability of a third generation synchrotron, this signal can be used as *I*_0_ for the measured scandium L-edge XAS spectra, even though the spectra and *I*_0_ were not measured simultaneously. The spectra were taken in step-by-step mode: 380–395 eV, Δ*E* = 1 eV; 397–415 eV, Δ*E* = 0.1 eV; 415–425 eV, Δ*E* = 0.75 eV. For the Sc L-edge spectra, only the TEY data were used. The main reason for choosing TEY is that the fluorescence measurements on thick pure samples are affected by overabsorption. This is especially critical when measuring the intense Sc L-edge spectra with soft X-rays, where already the non-resonant characteristic absorption depth is on the order of 50–100 nm (https://henke.lbl.gov/optical_constants/). As measuring XAS spectra in TEY mode is not affected by overabsorption, TEY is the preferred method.

The oxygen K-edge spectra were measured in both TEY and fluorescence mode, with no practical difference between the two modes. Here, we show data taken in TEY mode. These data were not normalized by *I*_0_, as the signal derived from the Cu plate showed an oxidation signature on the Cu surface, and hence cannot be used for normalization. We took *I*_0_ from the scattering signal from ScF_3_, which should be oxygen free. However, a small signature at the oxygen K edge was found, possibly stemming from oxygen contamination of ScF_3_ or from contaminations in the beamline. We tested the impact of this signature in *I*_0_ on the XAS spectrum, and found it irrelevant for the depth of analysis needed in this paper. Hence, here we show the raw, unnormalized O K-edge data.

## Results and discussion

### Structural analysis

**Fig. 1:**
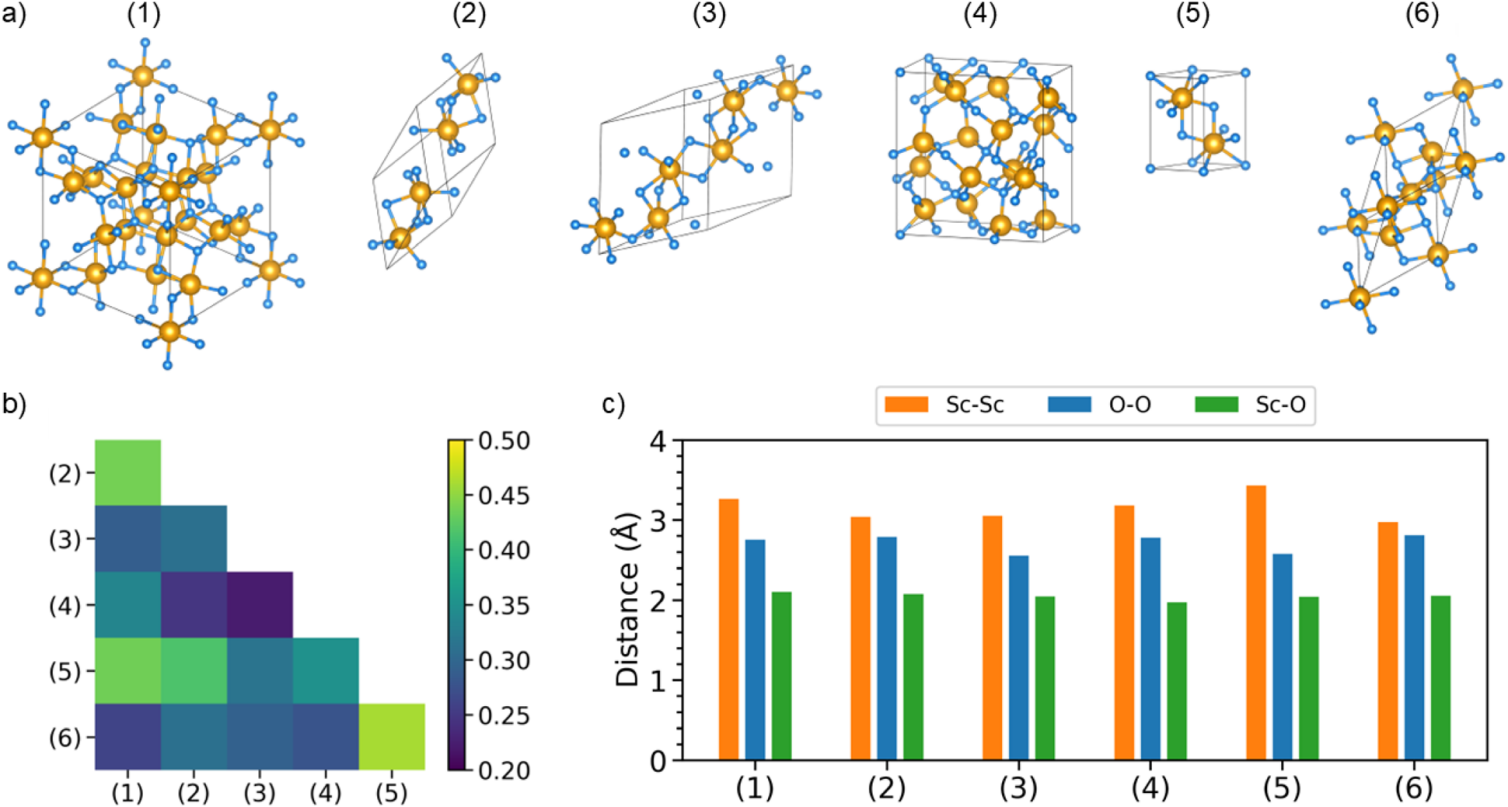
(a) Ball-and-stick representations, produced with the VESTA software,^52^ of the unit cells of the Sc_2_O_3_ polymorphs considered in this work, with Sc atoms depicted in gold and O atoms in light blue. (b) Similarity matrix among the considered Sc_2_O_3_ phases, labeled on both axes as Arabic numbers in parentheses, computed from the *F*-fingerprint metric (cosine distance): large (small) similarities correspond to low (high) values of *D*_cos_ in the displayed color bar. (c) Absolute values of the averaged interatomic Sc–Sc, O–O, and Sc–O distances within the considered Sc_2_O_3_ polymorphs.

We start our analysis by investigating the structural properties of the six Sc_2_O_3_ polymorphs considered in this work (Fig. 1a). Their geometries are available in the Materials Project Database;^49^ see identification numbers (entry-ID) in Table 1. Among the considered crystals, the cubic bixbyite phase, labeled as (1), is considered the most stable one, although the monoclinic polymorph (3) and the trigonal (or hexagonal) phase with space group *P*3̄*m*1, herein indicated as phase (6), can also be formed under ambient conditions.^20–22^ Phase transitions among these phases upon increasing pressure have been reported in the literature.^20,50^ To the best of our knowledge, the remaining structures (2), (4), and (5) have only been computationally predicted so far. However, since they may appear as local phases of polycrystalline samples or be stabilized under different thermodynamic conditions, it is worth including them in this study and analyzing their characteristics in comparison with the experimental cubic polymorph.

We assess structural similarities among the considered Sc_2_O_3_ phases employing the *F*-fingerprint method introduced by Oganov and Valle.^51^ Using the cosine distance metric,5
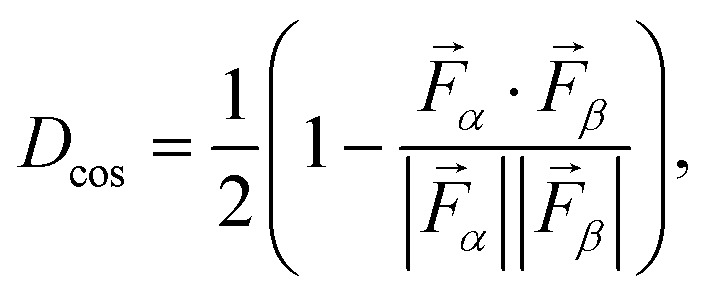
where 
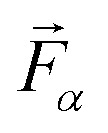
 and 
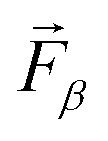
 are high-dimensional vectors (the so-called *fingerprint* vectors) encoding the radial distribution functions of each atomic pair in each crystal, this technique enables a straightforward comparison between two different phases *α* and *β*. The results of this analysis, displayed in Fig. 1b, point to pronounced similarities between the experimentally known cubic (1) and trigonal (6) polymorphs. Analogies with these experimental phases appear for the other synthesized monoclinic crystal (3) as well as for the computationally predicted orthorhombic crystal (4). On the other hand, the remaining trigonal phases (2) and (5) depart significantly from the cubic structure. Among the computationally predicted phases, we recognize similar fingerprints between phases (4) and (2), as well as with the monoclinic and trigonal high-pressure crystals (3) and (6). Polymorph (5) shows the least similarities with the other structures, as indicated by the relatively high values of the corresponding cosine metric (light green/yellow in Fig. 1b).

Interatomic distances provide another crucial point of comparison for the structural properties of Sc_2_O_3_ polymorphs. The corresponding results, obtained by averaging the individual Sc–Sc, O–O, and Sc–O bond lengths in each compound (Fig. 1c), largely confirm the trends from the *F*-fingerprint analysis. However, some important differences warrant closer inspection. The cubic phase (1) exhibits an average Sc–O bond length of 2.1 Å, in excellent agreement with experiments,^17,50,53^ while the Sc–Sc and O–O separations are 3.3 Å and 2.7 Å, respectively, consistent with its highly symmetric coordination. The computationally predicted orthorhombic polymorph (4) shows almost identical interatomic distances to the cubic phase (1), although its O–O bonds are slightly more extended while the Sc–O and Sc–Sc ones are shorter. Furthermore, structure (4) displays the shortest average Sc–Sc (3.2 Å) and O–O distances (2.3 Å) due to its relatively compressed unit cell (see Fig. 1a). The monoclinic phase (3) is characterized by systematically shorter interatomic distances than the cubic one. In contrast, the trigonal phase (6) exhibits significantly shorter Sc–Sc bonds compared to the cubic polymorph, despite having slightly elongated Sc–O bonds up to 2.3 Å and a more expanded Sc–Sc separation of 3.7 Å.

Overall, the Sc–O distances are mostly insensitive to the specific crystal structure. This result is consistent with the consideration that the cation–anion bond should be affected the least by the overall atomic arrangement in the lattice, depending on its symmetry. Following the same line of reasoning, O–O and especially Sc–Sc distances are instead more influenced by the specific atomic coordination due to the crystal structure. While the *F*-fingerprint analysis reveals close structural resemblance between the experimental cubic and hexagonal polymorphs (1) and (6), as well as between the computationally predicted trigonal and orthorhombic phases (2) and (4), the monoclinic and orthorhombic polymorphs generally exhibit broader distributions of distances, reflecting their lower symmetry. Finally, the monoclinic structure (3) and the trigonal lattice (5) display more pronounced deviations in terms of bond lengths, as a signature of very different local motifs and/or packing densities.

### Electronic properties

**Fig. 2:**
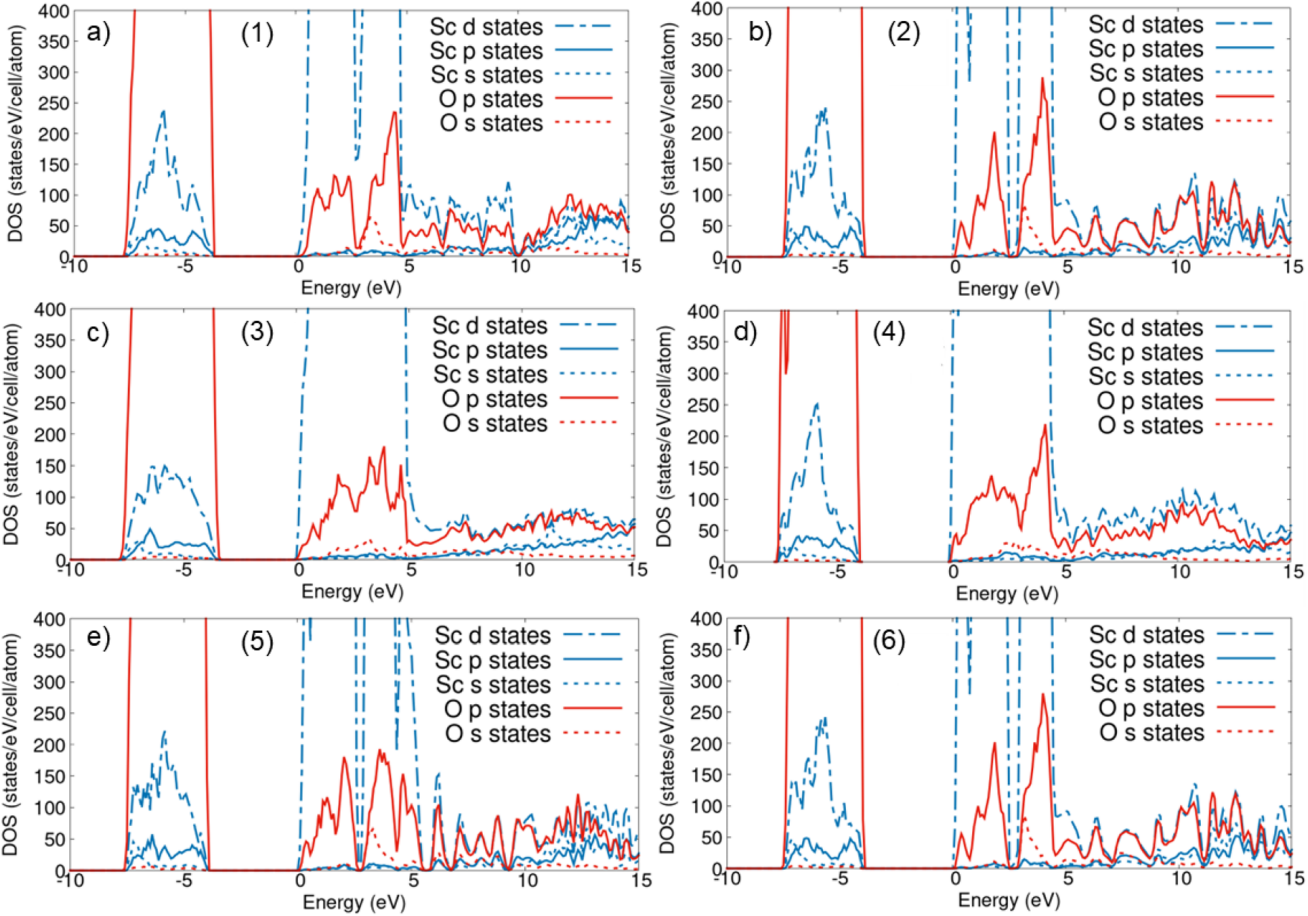
Atom-resolved PDOS of (a) the experimentally resolved cubic phase (1) and the computationally predicted (b) trigonal (2), (c) monoclinic (3), (d) orthorhombic (4), and trigonal phases (e) (5) and (f) (6) of Sc_2_O_3_. The Fermi energy is set to zero at the bottom of the conduction band of each crystal. The conduction band minimum is set to 0.0 eV in all plots. A Gaussian broadening of 100 meV is adopted for visualization.

In the next part of our study, we focus on the electronic properties of the Sc_2_O_3_ polymorphs as a basis for the subsequent analysis of the XANES spectra. To this end, we inspect the atom-resolved PDOS displayed in Fig. 2. Our results consistently indicate that all crystal phases are semiconducting with band gaps of the order of 4 eV, in agreement with earlier DFT predictions adopting comparable settings.^2,54,55^ In another DFT work, the band gap of the high-pressure monoclinic phase (3) was reported to be ∼10% larger than the cubic one.^21^ This trend is reproduced by our findings (compare Fig. 2a and d). All our results systematically underestimate the experimental gap by about 2 eV,^56,57^ as expected from the adopted semi-local approximation for the exchange-correlation functional. Range-separated hybrid functionals lead to much more accurate band-gap predictions for Sc_2_O_3_.^55^

A close inspection of the PDOS reveals that the valence and conduction bands of the six considered Sc_2_O_3_ polymorphs carry the same atomic orbital contributions, as expected from their identical stoichiometries (Fig. 2). The uppermost valence region is dominated by the O 2p-states hybridized with the single electron state in the Sc 3d-shell.^2,54^ The lowest conduction band hosts the remaining unoccupied Sc 3d levels partially hybridized with O 2p orbitals. Differences due to the different packing arrangements emerge in the distribution and relative weight of the orbital contributions in the PDOS, especially in the unoccupied region between 0 eV (where the conduction-band minimum is set) and 5 eV. In this range, the experimental cubic phase (1) exhibits two energetically separated sub-bands with hybridized Sc d- and O p-character (Fig. 2a), identified also in previous work.^2,55^ This two-feature structure is a direct result of the crystal field splitting of the Sc-3d states, dictated by the local Sc–O coordination environment. Although the corresponding values reported in Fig. 1c are similar across all polymorphs, in the trigonal phases (2), (5), and (6), the atomic arrangement enhances this band separation, leading to a more pronounced dip in the PDOS compared to the other structures with cubic, monoclinic, or orthorhombic symmetries.

The higher-energy sub-band includes a non-negligible O s-contribution, which remains non-zero, although weak, throughout the entire range visualized in Fig. 2. The computationally predicted polymorphs (2) and (5) exhibit similar features to the trigonal phase (6), except for slight changes in the relative height of the PDOS peaks (Fig. 2b, e and f). The monoclinic phase (3) and the predicted orthorhombic polymorph (4) are characterized by a continuous unoccupied band up to 5 eV dominated by Sc d- and O p-states (Fig. 2c and d). At higher energies, the density of states is significantly reduced in all polymorphs. The electronic states are again hybridized between Sc d- and O p-electrons, with a slight predominance of the former between 5 and 10 eV in the cubic phase (1), as shown in Fig. 2a. In the same energy window, all other phases present almost equal contributions between these two states. The similarities highlighted by the PDOS analysis are generally consistent with those that emerged for the structural properties. In particular, the cubic polymorph has structural and electronic characteristics very close to those of the high-pressure trigonal phase (6). In contrast, the monoclinic phase (3) exhibits analogous features to the orthorhombic crystal (4).

### Core spectroscopy

We continue our analysis with the XANES spectra calculated from the solution of the BSE.^27,44^ Due to the high computational costs of these simulations, we exclude the experimental cubic crystal (1) and the computationally predicted orthorhombic polymorph (4), both characterized by large unit cells hosting 40 atoms. However, based on our prior analysis of the structural and electronic properties of the different phases, we found that the experimental cubic phase (1) is very similar to the simulated trigonal phases, particularly phase (6). Similarly, the excluded orthorhombic phase (4) shares key characteristics with the monoclinic crystal (3). Given the strong similarities, we expect the analysis of the smaller, computationally accessible polymorphs (2), (3), (5), and (6) to provide valuable, transferable information for understanding the XANES characteristics of the larger systems (1) and (4). We consider excitations from both the O K-edge and Sc L_2,3_-edge. The former are given by transitions from the O 1s electrons, while the latter are from the Sc 2p core states of both spin multiplicity 
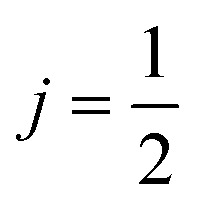
 and 
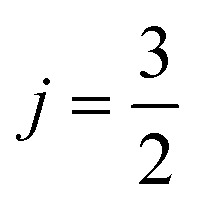
, corresponding to the L_2_ and L_3_ sub-edges, respectively.^58^ In this analysis, we contrast the results of our calculations with corresponding experimental spectra obtained for the available Sc_2_O_3_ sample.

### O K-edge

**Fig. 3:**
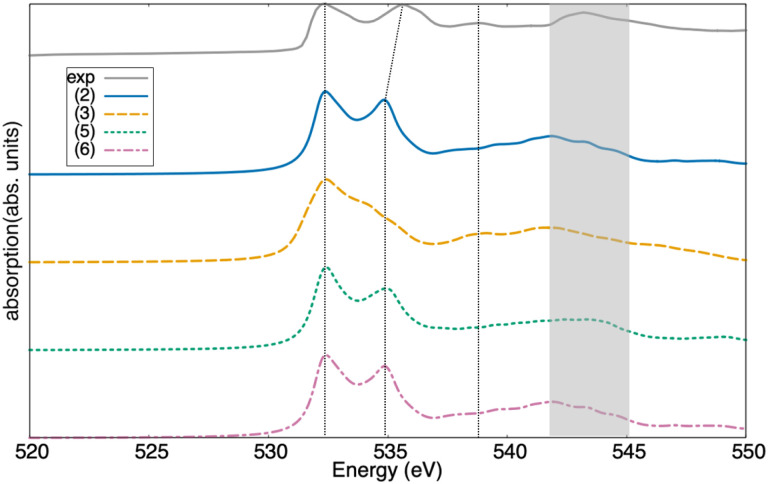
O K-edge XANES spectra obtained from experiment (gray solid curve) and computed from the BSE for the computationally predicted trigonal phase of Sc_2_O_3_ with the *R*3̄*c* space group (2), the high-pressure monoclinic polymorph (3), the computationally predicted trigonal phase with space group *R*3̄ (5), and the experimental trigonal phase appearing at high-pressure (6). Vertical bars mark the energy of the main resonances in the experimental spectrum, connecting them with the corresponding features in the computed results. The high-energy maximum between 541.5 and 545 eV is highlighted by a shaded gray rectangle.

The O K-edge XANES of the (2), (3), (5), and (6) Sc_2_O_3_ polymorphs computed from the BSE are reported in Fig. 3 along with the data measured at the synchrotron. The experimental spectrum exhibits a two-peak structure at the onset, followed by broader maxima at higher energies. These features are well captured by the XANES computed for the two trigonal phases, both the predicted (2) and the experimental one (6) (Fig. 3). This agreement validates our initial hypothesis derived from the structural and electronic similarities, allowing phases (2) and (6) to serve as effective models for the excluded cubic phase (1). These calculations reproduce quite well the relative intensity of the first two peaks, while slightly underestimating their energy separation. This underestimation is related to the uniform scissors shift used to align the calculated spectrum to the experimental one, which does not account for state-dependent variations of the self-energy correction. The broader, higher-energy features are also well reproduced by the BSE spectra. From a detailed comparison of the spectra in Fig. 3, we notice that phase (3) exhibits distinct features, while phases (2), (5), and (6) are not easily distinguishable based on the O K-edge features alone, particularly as the second excitation and the higher-energy absorption remain largely aligned across these three polymorphs.

The XANES computed for the remaining computationally predicted trigonal structure (5) is also in fair agreement with the experiment, especially concerning the higher energy maximum at ∼543 eV (Fig. 3). In contrast, the second peak at the onset is significantly underestimated in intensity, and its energy is still too low compared to the experiment. Finally, the XANES computed for the monoclinic phase (3) mostly differs from our experimental data recorded for the cubic phase (see Fig. 3). This discrepancy is expected from its distinct electronic-structure features discussed in Fig. 2c. In this case, the second peak at the onset is completely missing, being replaced by a shoulder of the first one. Moreover, at higher energies, the maximum centered at ∼543 eV is red-shifted, and the oscillator strength above 545 eV is drastically dropping.

**Fig. 4:**
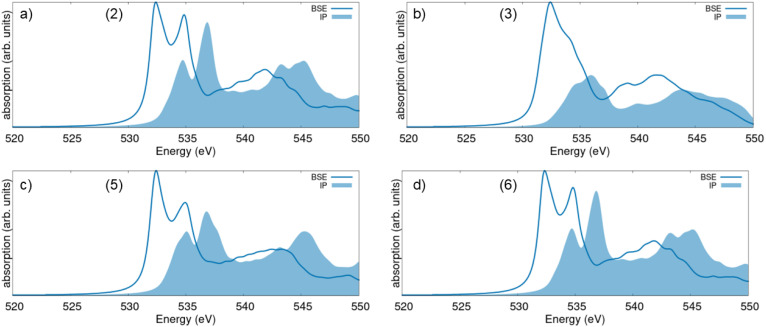
O K-edge XANES spectra computed from the solution of the BSE (solid line) and in the IPA (shaded area) for (a) the computationally predicted trigonal phase of Sc_2_O_3_ with the *R*3̄*c* space group (2), (b) the high-pressure monoclinic polymorph (3), (c) the computationally predicted trigonal phase with space group *R*3̄ (5), and (d) the experimental trigonal phase appearing at high-pressure (6).

To better understand the origin of the spectral features appearing in the XANES computed from the BSE, we contrast these results with the IPA, where electron–hole correlations are neglected. IPA spectra are expected to carry the PDOS signatures, providing direct insight into the origin of the peaks. A glance at Fig. 4 reveals a significant discrepancy between the BSE and IPA results, implying relevant excitonic effects in the O K-edge XANES of all the examined Sc_2_O_3_ phases. The two-peak structure at the onset of both the BSE and IPA spectra stems directly from the lowest-energy window of the unoccupied O PDOS region (Fig. 2). However, while in the IPA result the relative weight of the two peaks resembles the O p-orbital contributions to the PDOS, in the BSE spectrum, the lowest-energy peak has a relatively larger oscillator strength, as discussed above. Analogous characteristics are found in the O K-edge XANES of other sesquioxides, like β-Ga_2_O_3_ ^13,59^ and γ-Al_2_O_3_,^60^ hinting to a general trend in the XAS of this class of materials that is worth of deeper, dedicated investigation in the future.

In the XANES computed for the monoclinic polymorph (3), the two peaks at the onset are not well resolved with the adopted broadening of 0.5 eV (Fig. 4b). Similar to the BSE result, the IPA spectrum exhibits a broad maximum at the onset, reflecting in turn the shape of the lowest unoccupied O band in the PDOS (Fig. 2c). Excitonic effects significantly redshift the absorption maxima by about 2 eV, corresponding to the exciton binding energy. Moreover, electron–hole correlations enhance the oscillator strength of the lowest-energy peak by approximately a factor of 3 compared to the IPA spectrum (Fig. 4c). The pronounced red shift of the spectral weight in the O K-edge XANES of the considered Sc_2_O_3_ polymorphs leads to a relative decrease of the oscillator strength of the higher-energy, broad maximum. Except for the monoclinic phase (3), where the absorption band between 538 and 542 eV in the BSE spectrum is stronger than its (blue-shifted) counterpart in the IPA result (Fig. 4c), in all the other XANES, this absorption region loses intensity upon inclusion of electron–hole interactions.

### Sc L_2,3_-edge

**Fig. 5:**
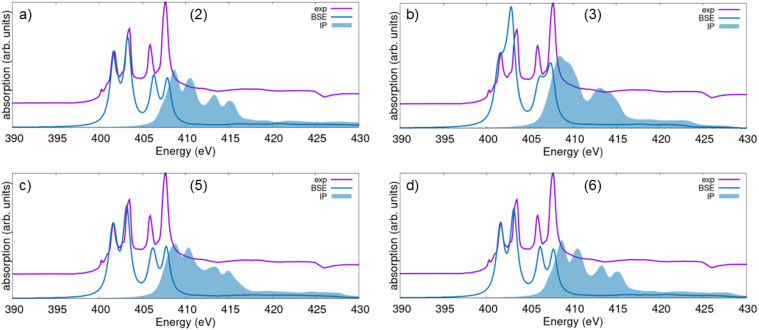
Simulated XANES spectra, both from the solution of the BSE (solid lines) and in the IPA (shaded area) from the Sc L_2,3_-edge for (a) the computationally predicted trigonal phase of Sc_2_O_3_ with the *R*3̄*c* space group (2), (b) the high-pressure monoclinic polymorph (3), (c) the computationally predicted trigonal phase with space group *R*3̄ (5), and (d) the experimental trigonal phase appearing at high pressure (6). Measured and computed spectra are aligned to the intensity of the first resonance. The experimental result (violet curve) is offset along the *y*-axis to facilitate comparison.

We complete our analysis by inspecting the Sc L_2,3_-edge XANES of the four considered Sc_2_O_3_ phases. In this case, we show in a single graph both theoretical results from BSE and the IPA, contrasting them against our experimental data, which are in turn consistent with an earlier measurement of the total electron yield.^17^ The XANES spectra displayed in Fig. 5 exhibit the typical signatures of L_2,3_-edge spectra of light metallic elements, with two manifolds of two-peak structures at the onset due to transitions from the 2p_1/2_ and 2p_3/2_ electrons of Sc atoms separated from each other by a few eV in energy. This spectral shape resembles that expected for 3d^0^ systems,^61–65^ where the main features arise from transitions into the unoccupied 3d conduction band, split by crystal field effects. The BSE results obtained for the trigonal polymorphs (2), (5), and (6) are in excellent agreement with the experiment as far as the lowest-energy sub-edge (L_3_) is concerned (Fig. 5a, c and d). The higher-energy peaks, associated with the L_2_ sub-edge, are correctly positioned in energy, but their relative intensity is severely underestimated. This is a well-known shortcoming of these calculations as implemented in the LAPW + lo formalism of the exciting code,^27,44^ which requires extremely large energy cutoffs for the local field effects, beyond our current computational capabilities, to correctly reproduce the L_3_–L_2_ branching ratio. Similar issues also appear in reproducing the pre-edge features that are present in the experimental spectrum, around 400 eV (Fig. 5). These signals are due to the interplay of complex mechanisms involving atomic exchange, crystal field splitting and other solid-state effects, and require extraordinary computational efforts to be accurately resolved with multipurpose BSE implementations like the one in exciting. Optimized BSE developments for XAS, such as the one proposed by Shirley,^66^ offer higher accuracy and efficiency, especially for computing spectra of 3d^0^ systems.^63^

A closer inspection of Fig. 5 reveals that the best agreement with the experimental result is obtained for the BSE spectrum of the computationally predicted trigonal polymorph (2). This excellent match, where the relative intensity and energy of the two peaks in the L_3_ sub-edge almost perfectly mirror the measurement (Fig. 5a), is consistent with our prior finding that phase (2) shares strong structural and electronic similarities with the experimental cubic phase (1) that was excluded from the BSE set due to its large size and consequently computational costs. While the experimentally adopted TEY mode is generally surface-sensitive, our sample consists of Sc_2_O_3_ powder. The measured spectrum is therefore an average over various surface planes and all polarization angles, minimizing the impact of specific, polarization-dependent surface reconstructions, as reported by Himpsel *et al.* for Ca-based samples.^67^ The excellent agreement between the calculated spectrum of phase (2), assuming the sample as an idealized bulk crystal, and the experimental L_3_ line shape strongly indicates that the measured XANES signal is primarily dominated by the bulk electronic structure with negligible contributions from surface effects.

In the simulated XANES of the other trigonal phases (5) and (6), a slight mismatch in oscillator strength and energy can be seen in Fig. 5c and d. Similar to the O K-edge spectrum, also for the Sc L_2,3_-edge XANES, the result obtained for the monoclinic phase (3) is the furthest from the experiment (Fig. 5b). This lack of agreement confirms that this phase is structurally, electronically, and spectroscopically different from the cubic sample. This distinction further supports our PDOS and structural analysis, suggesting that phase (3) is instead the best representative for the orthorhombic phase (4), which was excluded from this analysis due to its large size. In both sub-edges, the two-peak structure is only hinted at, but not as well resolved as in the measurement. Moreover, the relative energies of the maxima are not well reproduced, confirming that this Sc_2_O_3_ phase is not only structurally (Fig. 1a) but also electronically and spectroscopically very different from the cubic one.

Comparison between the spectra calculated from the BSE and in the IPA reveals relevant excitonic effects also at the Sc L_2,3_-edge (Fig. 5), in analogy with the O K-edge XANES (Fig. 4). Here, they manifest through a substantial red shift of the absorption onset on the order of 5–8 eV, depending on the specific polymorph. The relative oscillator strength between the two sub-edges is similar between BSE and IPA but in disagreement with the experiment, as discussed above. However, within the lowest-energy sub-edge (L_3_), we notice a redistribution of the spectral weight toward the second sub-peak when electron–hole correlations are taken into account. This is another expected characteristic of these XANES simulations at the L_2,3_-edge of light metallic elements.^27,30,36,68^

From the analysis of the IPA spectra, we can identify the electronic contributions to the peaks in the XANES. Selection rules enable active transitions from 2p electrons of Sc to unoccupied bands with both s- and d-character. Comparison with Fig. 2 reveals that the latter contributions dominate the bottom of the conduction region of all polymorphs, consistent with the fact that Sc has only one occupied state in its 3d-shell. The characteristic two-peak structure of the 3d-electronic states in the conduction band of (2), (5), and (6) Sc_2_O_3_ phases (see Fig. 2b, e and f) is consistent with the corresponding structure observed in both XANES sub-edges (Fig. 5a, c and d). Likewise, the more compact structure of this band manifold in polymorph (3) is reflected in both its IPA and BSE spectra (Fig. 5b).

## Summary and conclusions

In summary, we presented a combined experimental and first-principles study on the X-ray absorption properties of Sc_2_O_3_. Experimental XANES data collected for the most stable cubic phase are contrasted against *ab initio* results computed for six crystalline polymorphs, including the experimentally known cubic, monoclinic, and trigonal phases (the last two appearing at high pressure), and three computationally predicted structures with trigonal and orthorhombic symmetry.

Our analysis first focused on the structural similarities among the considered phases. We found remarkable analogies between the stable cubic polymorph and the two trigonal structures. These similarities are consistently reflected in the electronic structure of the materials, as revealed by their atom-projected density of states, providing essential information for mapping the transitions from the core electrons (O 1s and Sc 2p) to unoccupied target states. The BSE results, obtained for a computationally accessible subset of the original pool of structures, are in very good agreement with the measurement, validating our strategic approach of adopting the spectra calculated for smaller polymorphic analogues using the state-of-the-art BSE approach. A direct comparison with results computed in the IPA, whereby electron–hole correlations are neglected, highlights the importance of including excitonic effects for properly describing core-level excitations in Sc_2_O_3_. Excitonic effects notably impact the O K-edge spectra by red-shifting the overall spectral weight by a couple of eV and by additionally swapping the relative intensity of the two peaks at the onset. This behavior is similar to other transition-metal sesquioxides like β-Ga_2_O_3_ ^13,59^ and γ-Al_2_O_3_.^60^ Our results reproduce well the strongest resonances in the Sc L_2,3_-edge XANES, particularly the relative intensity of the characteristic two peaks at the onset. However, we acknowledge their current limitation in capturing weak pre-edge features and the L_2_–L_3_ branching ratio, which require considerably larger energy cutoffs for local field effects that are currently beyond our computational capabilities.

Our comprehensive analysis of the XANES from the O K-edge and Sc L_2,3_-edge offers important insight into the spectral fingerprints of the high-pressure monoclinic (3) and trigonal (6) phases. While the latter exhibits very similar characteristics to the cubic phase, the monoclinic polymorph presents distinctly different spectral features, in line with its unique structural and electronic properties, which could favor its identification upon phase transition during *in situ* experiments. The significant similarities among the various Sc_2_O_3_ phases suggest an important caveat: distinguishing between polymorphs with subtle structural and electronic differences may be difficult experimentally. However, the fact that we can differentiate between the cubic phase and its trigonal analogues based on small shifts in L_3_ peak splitting and O K-edge features demonstrates that combining information from both edges does enable robust, if challenging, discrimination. Moreover, the successful use of smaller, electronically analogous polymorphs to validate our method suggests a viable strategy for accurately predicting XANES characteristics of complex, large-unit-cell materials at affordable computational costs using *ab initio* many-body methods. This is crucial not only for accurate sample characterization but also for the development of robust datasets feeding emerging machine-learning and other artificial-intelligence-based models used for XANES analysis.^69–71^ Adopting more advanced synchrotron techniques, such as (resonant) inelastic X-ray scattering at the metal-hybridized O K-edge features or non-resonant Sc L-edge X-ray emission spectroscopy, may contribute to improving the characterization of Sc_2_O_2_ polymorphs for improved sample diagnostics.

## Conflicts of interest

There are no conflicts of interest to declare.

## Data Availability

The data presented in this work are available in the ZENODO repository at the following link: https://doi.org/10.5281/zenodo.16780711.
